# Characterization of normative fetal rhesus macaque brain development with magnetic resonance imaging

**DOI:** 10.1162/IMAG.a.160

**Published:** 2025-09-30

**Authors:** Joshua A. Karpf, Kara E. Garcia, Vergina C. Cuzon Carlson, Kathleen A. Grant, Jamie O. Lo, Christopher D. Kroenke

**Affiliations:** Division of Neuroscience, Oregon National Primate Research Center, Oregon Health and Science University, Beaverton, OR, United States; Department of Behavioral Neuroscience, Oregon Health and Science University, Portland, OR, United States; Department of Radiology & Imaging Sciences, Indiana University School of Medicine, Evansville, IN, United States; Division of Reproductive and Developmental Sciences, Oregon National Primate Research Center, Oregon Health and Science University, Beaverton, OR, United States; Divison of Maternal Fetal Medicine, Department of Obstetrics and Gynecology, Oregon Health and Science University, Portland, OR, United States; Advanced Imaging Research Center, Oregon Health and Science University, Portland, OR, United States

**Keywords:** gestation, neurodevelopment, fetal, magnetic resonance imaging, rhesus macaque, sexual dimorphism

## Abstract

Advances in motion correction magnetic resonance imaging methods have made it possible to track anatomical changes throughout the highly dynamic phase of fetal neurodevelopment. Characterizing the trajectory of normative brain development provides insight into the underlying biological processes driving growth, as well as a framework for identifying deviations that may be etiological markers of neurodevelopmental disorders. Rhesus macaques, which exhibit similar gestational neurodevelopmental timelines to humans, can be used to address the challenges of obtaining accurate longitudinal fetal imaging measurements and are a key preclinical resource for investigating experimental developmental perturbations. Additionally, the ability to examine biological factors including age and sex can provide important information regarding individual variability in development, but this is often precluded due to limitations in nonhuman primate samples, especially during gestation. To provide updated rhesus macaque magnetic resonance fetal templates allowing for the characterization of normative neurodevelopmental trajectories, we leveraged a unique, large mixed-longitudinal sample of 50 normally developing rhesus macaque fetuses (28 female and 22 male) scanned longitudinally and cross-sectionally *in utero* (105 scans) across the second half of gestation (post-conception gestational day (G) 85, G97, G110, G122, G135, G147, and G155; of a 165-day term). We generated anatomically segmented T_2_-weighted and mid-cortical surface templates at these ages, as well as a 4-year-old post-pubertal young adult template (10 female and 10 male) with corresponding fetal consistent anatomical segmentations for comparisons of fetal-to-adult values, which we provide to the neuroscience imaging community. In characterizing shape morphological features (surface area, curvature, and thickness) and volumetric brain development throughout the second half of gestation, we identify evidence of sexual dimorphism in rhesus macaque fetal brain growth and compare patterns of cortical development with findings from other species.

## 1 Introduction

*In utero* fetal magnetic resonance imaging (MRI) is increasingly being utilized as a clinical tool and research method for characterizing brain development, and advances in motion correction strategies as well as 3-dimensional volume reconstruction algorithms have enabled detailed characterization of brain growth beginning midway through human gestation (Nagaraj & Kline-Fath, 2022; Saleem, 2014; Studholme, 2011). MRI is safe during pregnancy (Tocchio et al., 2015) and can be performed on volunteer participants in clinical research contexts. Large sample T_2_-weighted fetal template images spanning the second half of gestation (Ciceri et al., 2024; Edwards et al., 2022; Gholipour et al., 2017; Kuklisova-Murgasova et al., 2011; Serag et al., 2012; Urru et al., 2023; Uus et al., 2023; Wu et al., 2021; Xu et al., 2022), along with publicly available analytic tools (Fidon et al., 2024; Rousseau et al., 2013; Urru et al., 2023) have aided in rapid advances to our understanding of fetal brain development. These efforts are mapping out a highly dynamic period in which the brain undergoes dramatic changes in size and shape, coincident with tissue cellular composition changes that result in changes in image contrast patterns seen in fetal brain MRI.

Rhesus macaques exhibit similar fetal development to humans (Barry et al., 2006; Liu et al., 2020; Malkova et al., 2006). In particular, similarities in brain anatomy make them an excellent preclinical resource for generating and validating interpretations of MRI-based measurements of fetal brain developmental dynamics. In studies of non-human primates (NHPs), it is possible to precisely control experimental conditions, which is important for establishing mechanistic links between characteristics of the intrauterine environment and their ensuing effects on brain development. In addition, highly accurate conceptional age dating is feasible in NHP studies, whereas pregnancy due dates estimated by last menstrual period and ultrasound in humans have limitations in accuracy. Gestation in the rhesus macaque is approximately 24 weeks (165 days) compared with 38.5 weeks (Jukic et al., 2013) (~270 days ovulation to birth) in humans. While macaques undergo slightly more extensive central nervous system development during gestation than humans (Sawada et al., 2012, 2014; Scott et al., 2016), the order of milestones in the gestational timeline is generally well conserved, including dynamic aspects of brain size and shape change over the second half of gestation (Habas et al., 2012; Shimony et al., 2016; Wang et al., 2017), as well as the associated changes in tissue image contrast (Saleem, 2014; Liu et al., 2020).

In order to provide an initial characterization of normative rhesus brain development over the second half of gestation, we have previously generated spatiotemporal templates of the fetal brain (Liu et al., 2020; Wang et al., 2017). Ethical and cost constraints preclude large number of animals analogous to currently ongoing population-scale human neuroimaging efforts (Casey et al., 2018; Edwards et al., 2022; Pfefferbaum et al., 2016; Volkow et al., 2024). Therefore, our approach has been to combine untreated control groups from multiple rhesus macaque pregnancy studies, and to complement these with smaller groups of animals to balance the demographics and time points sampled within the dataset to build a data resource that the neuroscience community can access. The Oregon National Primate Research Center (ONPRC) Fetal Macaque Brain Atlas (https://www.nitrc.org/search/?type_of_search=group&q=onprc) consists of brain images from 24 fetuses scanned 43 times at approximate gestational ages (G)85, G110, and G135 (Liu et al., 2020). Since building this resource, additional studies involving new groups of control animals have been performed, resulting in an increase in both the number of animals and gestational ages assessed. In order to ameliorate gaps identified at specific age ranges, we have also added MRI data from a specifically designed cohort, enabling us to expand the age range and frequency of template images to G85, G97, G110, G122, G135, G147, and G155, and these are referenced to a specifically generated post-pubertal template derived from 20 animals (~4.5 years old; 10 females). The fetal T_2_-weighted images were obtained from 105 *in utero* brain scans of 50 (28 female) fetal rhesus macaques. In this work, we utilize two recently implemented data analysis methods that have been applied to other species. Namely, nonlinear logistic regression is used to account for the sigmoidal increase in size of the brain and various sub-regions (Garcia et al., 2024, 2025), and development of the anatomical multimodal surface matching (aMSM) algorithm has facilitated regional analysis of growth of the cerebral cortical surface (Garcia, Robinson, et al., 2018; Garcia et al., 2024; Robinson et al., 2018). Normative trajectories using the extended dataset and analysis tools provide improved sensitivity to assess biological variables such as gestational age and fetal sex, providing new insights into comparative aspects of brain size and shape changes. The updated template, ONPRC Fetal Macaque Brain Atlas 2.0, is available to the community through the NeuroImaging Tools & Resources Collaboratory (NITRC) (https://www.nitrc.org/projects/onprc_fetal_2/).

## 2 Methods

### 2.1 Subjects

All experimental procedures utilized in this study were approved by the ONPRC Institutional Animal Care and Utilization Committee (IACUC) and conformed to the guidelines set forth in the *Guide for the Care and Use of Laboratory Animals*. Methods are reported in accordance with the ARRIVE guidelines (Kilkenny & Altman, 2010) (https://arriveguidelines.org). The ONPRC is accredited by the Association for Assessment and Accreditation of Laboratory Animal Care (AAALAC) International. The sample in this study was generated from scans performed at the ONPRC imaging core and includes control animals from previously published and unpublished datasets, composed of longitudinal cohort and cross-sectional designs collected between 2011 and 2024 (Table 1).

**Table 1.** Study demographics.

Group	Scan age	Animals	Females	Scans
Normative 1 (Liu et al., 2020; Wang et al., 2017)	G85, G110, G135	M1-M5 (5)	3	13
FASD (Jimenez et al., 2019; Liu et al., 2020; Wang et al., 2020)	G85, G110, G135	M6-M17 (12)	8	12
Protein Malnutrition (Karpf et al., 2024; Kirigiti et al., 2020; Liu et al., 2020; Lo et al., 2023; Roberts et al., 2018, 2021)	G85, G135	M18-M35 (18)	8	35
Fetal THC 1 (Ryan et al., 2024)	G85, G110, G135, G155	M36-M40 (5)	3	20
Fetal THC 2 (Shorey-Kendrick et al., 2025)	G110, G155	M41-M45 (5)	4	10
Normative 2	G97, G122, G147	M46-M50 (5)	2	15
Total fetal	G85-G155	50	28	105
Post-pubertal	(4.4 ± 0.44 years)	M51-M70 (20)	10	20

FASD, fetal alcohol spectrum disorder; THC, tetrahydrocannabinol; G, gestational age; M, monkey number.

In total, 105 scans of 50 rhesus macaque fetuses (N = 28 female, N = 22 male) generated from 40 dams at the ONPRC were conducted across the second half of gestation. For datasets obtained from investigations involving experimental manipulation, only “control” (non-treated) fetuses were included in this study. Specifically, monkeys (M) M1-M5 were part of a longitudinal study of normative cerebral cortical development (Liu et al., 2020; Wang et al., 2017), animals M6-M17, a cross-sectional study on the effects of maternal ethanol consumption during gestation (Jimenez et al., 2019; Liu et al., 2020; Wang et al., 2020), animals M18-M35, a longitudinal study of the effects of maternal protein reduction during gestation (Karpf et al., 2024; Kirigiti et al., 2020; Liu et al., 2020; Lo et al., 2023; Roberts et al., 2018, 2021), animals M36-M40, a longitudinal fetal study of gestational cannabis exposure (Ryan et al., 2024), animals M41-M45, a longitudinal fetal and postnatal study of gestational cannabis exposure (Shorey-Kendrick et al., 2025), and animals M46-M50, a longitudinal study of normative fetal and postnatal neurodevelopment (Supplemental Table S1). Apart from a subset of protein reduction study (animals M18-M29 which were socially group indoor/outdoor housed), dams were single- or pair-housed indoors on a 12-hr light/dark cycle. Dams were provided with standard monkey chow rations (TestDiet, St. Louis, MI) twice daily and given ad libitum access to water. Fetuses were excluded for fetal demise (2) or failed reconstruction due to poor image quality (1). In addition, a separate 4.4-year-old cross-sectionally collected sample of 20 young adult rhesus macaques (10 females) were included for male and female young adult comparisons. The post-pubertal animals were part of a study on alcohol use disorder and were single-housed (pair-housed for 2 hours each day).

### 2.2 Scan acquisition

The study sample includes historic data collected from 2011 to 2024 with slight variation in anesthetic procedures across subjects. Dams were sedated with ketamine (10–15 mg/kg IM), before being intubated, ventilated, and placed feet-first supine into the scanner. Anesthesia was induced with 2–3% isoflurane and maintained with 1–2% isoflurane vaporized in 100% O_2_ at a flow rate of ~2.0 L/min. Dams of animals M46-M50 additionally received a 0.2 mg/kg vecuronium bromide and 30–50 mg propofol bolus dose followed by intravenous infusion of 3 μg/kg/min vecuronium bromide and 180–240 μg/kg/min propofol (isoflurane reduced to ~0.4%), and fetal brain scans were performed under expiratory breath holds to minimize fetal motion. Animals were kept warm with a Bair Hugger, blankets, and circulating water blanket. Scans were collected using a Siemens 3T Tim Trio or PRISMA paired with a quadrature transmit, 15-channel receive human “extremity” RF coil (QED, Cleveland, OH). Vital signs including end-tidal carbon dioxide, respiration rate, pulse oximetry, non-invasive blood pressure, and O_2_ saturation of the dams were monitored during the entirety of the scans. Following a localizer to identify fetal head position, 3 repetitions of Turbo Spin-Echo (TSE) T_2_-weighted images were acquired as 1 mm thick, 2D image stacks with 0.66 mm^2^ in-plane resolution, 5000 ms repetition time, 97-ms echo time, echo train length of 27, 120° flip angle, a generalized auto-calibrating partially parallel acquisition (GRAPPA) factor of 2, and bandwidth of 362Px/Hz or 3 repetitions of Half-Fourier Acquisition Single-shot Turbo spin-Echo (HASTE) T_2_-weighted images acquired as 1 mm thick, 2D image stacks with 0.5–0.64 mm^2^ in-plane resolution,1200–1700 ms repetition time, 102–116 ms echo time, echo train length of 256, 150° flip angle, a generalized auto-calibrating partially parallel acquisition (GRAPPA) factor of 2, and bandwidth of 781 Px/Hz were collected in each of the 3 maternal cardinal axes (axial, sagittal, and coronal). Scans were clustered around specific study-dependent time points at G85, G97, G110, G122, G135, G147, and/or G155 (all ±7 days; rhesus term is ~165 days). Thus, 7 gestational templates were generated for analysis. For the comparative late adolescent/young adult sample, 20 animals (10 female) mean age 4.4 ± 0.44 years were sedated and anesthetized as described above and scanned with a 16-channel pediatric head coil (QED, Cleveland, OH). Two 3D T_2_-weighted sampling perfection with application-optimized contrasts using different flip angle evolution (SPACE) series were acquired with 0.5 mm isotropic resolution, 3200 ms repetition time, 385 ms echo time, 320 x 320 x 324 image matrix, and 120° flip angle.

### 2.3 Volumetric image processing and template generation

3D volumes with isotropic resolution equal to the acquired in-plane resolution were constructed using the BTK toolkit (Rousseau et al., 2013) or 0.5 mm isotropic resolution using iterative slice profile deconvolution (Fogtmann et al., 2013) for *in utero* HASTE scans. For adult acquisitions, the two SPACE volumes were merged to create a single, signal-averaged volume. Reconstructed fetal and merged adult volumes were then processed identically. Brain masks were generated semi-manually using ITK-SNAP (Yushkevich et al., 2006), and volumes were bias corrected (Tustison et al., 2010) prior to skull stripping. The resulting brain volumes were intensity normalized and rescaled brains were rigidly aligned to the ONPRC18 template (Weiss et al., 2021). Brain templates were generated using Advanced Normalization Tools (ANTs) (Avants et al., 2009, 2011) buildtemplateparallel function with default settings: 0.25 gradient step size, 4 template iterations, 30 x 50 x 20 max iterations in each registration, Greedy-Syn transformation, and the cross-correlation metric. Template anatomical segmentations were adapted from existing fetal segmentations (Liu et al., 2020; Ryan et al., 2024) and manually corrected for each age template. Regions were parcellated as they became apparent across development. From G85 to G155, the external (extra-axial) cerebrospinal fluid (CSF), cortical plate, subplate, ventricles, thalamus, cerebellum, brainstem, and corpus callosum were readily identifiable and segmented. The germinal and fibrous matrix layers (GMAT) were identifiable in the G85, G97, and G110 images, and the caudate, hippocampus, amygdala, and hypothalamus were identifiable beginning at G97. It is important to note that at G85, tissue that will become the amygdala, hippocampus, and hypothalamus is likely being classified as germinal matrix/cortical plate and thalamus, respectively. At G85, only the nascent striatum could be parcellated, and at G97 and G110, the lentiform nucleus could be differentiated from the caudate, but was without a clear border between the putamen and globus pallidus. Beginning at G122, the striatal distinction between the caudate, putamen, and globus pallidus was possible (Table 2). Individual brain volumes were registered to the corresponding age study-specific template, and the associated template segmentation label maps were applied. Regions were combined (identifiable at all seven ages) into the following parcellations for analysis: whole brain, (parenchymal and ventricular volume excluding extra-axial CSF), extra-axial CSF, cortical plate, subplate, subcortical gray matter, brainstem, and cerebellum. Volumes were calculated by individually masking labeled regions and multiplying the number of masked voxels by the voxel volume.

**Table 2.** Template tissue categories.

Tissue categories			G85	G97	G110	G122	G135	G147	G155
1. Cerebrospinal fluid			✔	✔	✔	✔	✔	✔	✔
2. Cortical plate			✔	✔	✔	✔	✔	✔	✔
3. Subplate			✔	✔	✔	✔	✔	✔	✔
4. Ventricles			✔	✔	✔	✔	✔	✔	✔
5. Germinal and fibrous layers			✔	✔	✔				
	103. Hippocampus			✔	✔	✔	✔	✔	✔
	106. Amygdala			✔	✔	✔	✔	✔	✔
6. Thalamus			✔	✔	✔	✔	✔	✔	✔
	107. Hypothalamus			✔	✔	✔	✔	✔	✔
7. Cerebellum			✔	✔	✔	✔	✔	✔	✔
8. Brainstem			✔	✔	✔	✔	✔	✔	✔
9. Corpus Callosum			✔	✔	✔	✔	✔	✔	✔
10. Striatum			✔						
	101. Lentiform nucleus			✔	✔				
		101. Putamen				✔	✔	✔	✔
		104. Globus pallidus				✔	✔	✔	✔
	102. Caudate			✔	✔	✔	✔	✔	✔

### 2.4 aMSM surface processing

Mid-cortical surfaces were generated from template and individual segmentations using MATLAB (MathWorks, Inc., Natick, MA) as previously described (Garcia et al., 2024). Briefly, the brainstem, cerebellum, and extra-axial CSF were removed from right and left hemisphere segmentations and remaining non-cortical plate voxels were converted to a binary mask. Cortical plate voxels closer to masked voxels than the outside of the brain were added to the binary mask and remaining cortical plate voxels were removed. The edited mid-cortical masks were then smoothed using the Matlab “smooth3” function with a 5-voxel “box” smoothing kernel. Surface meshes were created using the MATLAB function “isosurface” from the smoothed binary masks, corresponding to the binary mask values of ≥0.5. Hemispheric surface areas were computed using the connectome workbench functions “-surface-vertex-areas” and “metric-stats.” Normalized or non-dimensionalized curvature (product of mean curvature and characteristic radius of the brain, i.e. the square root of cortical surface area divided by 4π) was calculated using connectome workbench. Thickness was calculated as the ratio of cortical plate volume to mid-cortical surface area. Template surfaces were aligned using the aMSM algorithm (Garcia, Robinson*,* et al., 2018; Robinson et al., 2018) to assess regional cortical surface area expansion. This algorithm is a surface registration technique that utilizes surface data (such as mean curvature, used in this study) as well as the minimization of surface strain energy resulting from the deformation between surfaces to drive surface matching in order to achieve physically plausible point correspondence between surfaces (Garcia, Kroenke, et al., 2018; Garcia et al., 2024, 2025). Forward and reverse expansion direction output was averaged prior to overlaying the corresponding age hemispheric surfaces. The repository of resources needed to perform aMSM analyses can be found at https://github.com/KEGarciaLab.

### 2.5 Analysis and statistical modeling

A model selection procedure was followed to determine an appropriate analytical expression to represent the nonlinear changes in brain region size with gestational age for the various brain regions. Total regional brain volume (or in some cases, surface area, thickness, or curvature), V, was computed at each age, t, for each fetus, as listed in Supplemental Table S1. For each region, the expression for V(t) with the lowest Bayesian information criterion (BIC) was selected as the model that provides the closest approximation to the data without incorporating an excessive number of adjustable parameters, among the following set. A linear model consists of adjustable parameters for a slope (m) and value at t=0 (b)

V(t)=mt+b.

A quadratic function, with adjustable parameters consisting of the coefficients $c_{0}$, $c_{1}$, and $c_{2},$ was also applied to each dataset

$$V\left(t\right)=c_{2}t^{2}+c_{1}t+c_{0}$$.

To approximate the sigmoidal change in size typically associated with growth processes, a three-parameter logistic expression was fitted to the data

$$V\left(t\right)=A/\left(1+\text{exp}\left(-R_{g}\left(t-t_{mid}\right)\right)\right)$$.

The three adjustable parameters include the asymptotic limit, A, the timing of the inflection point between accelerating and decelerating volume expansion, $t_{mid}$ , and the maximal rate of expansion, $R_{g}$, achieved at $t_{mid}$ . These parameters are described in detail as they relate to MRI-derived characterization of brain growth in Garcia et al. (2024). The three-parameter logistic curve is constrained to a value of 0 when t=0 . This constraint can be relaxed with a four-parameter logistic expression, which is equal to B at t=0

$$V\left(t\right)=\left(A-B\right)/\left(1+\text{exp}\left(-R_{g}\left(t-t_{mid}\right)\right)\right)+B.$$

Last, a Gompertz function is another frequently utilized analytical expression to describe growth processes. Compared with the logistic expression, a Gompertz curve can more accurately approximate processes that involve a rapid acceleration phase, and a protracted decelerating rate of size increase

V(t)=a*exp(−b*exp(−c*t)).

The adjustable parameters for the Gompertz function a, b, and c can be converted into an inflection point between accelerating and decelerating volume expansion, $t_{mid}^{G}$ (the value of t in which the second derivative of V equals 0), $t_{mid}^{G}=ln\left(b\right)/c$ , and the maximal rate of growth $R_{g}^{G}$, (the first derivative of V, evaluated at $t_{mid}^{G}$), $R_{g}^{G}=ac/e$ , with e being Euler’s number.

A statistical analysis framework that accounts for the repeated measurements on a subset of the fetuses studied here was desired for characterizing potential sex-dependent effects or other potentially influential factors. However, it was observed that growth of all examined brain structures exhibited a nonlinear dependence on gestational age (as described in Results). Therefore, the model selection procedure was applied as described above to account for the nonlinear effect of age, and then a linear mixed effects model (lme4 package in R (Bates et al., 2015)) was used to assess for other statistically significant effects such as fetal sex by analyzing the residuals following the nonlinear analysis. Specifically, mixed-effect models considering the interaction between sex and age as fixed effects, with fetus as a random effect, were used to analyze sex differences for volumes and surface metric residuals according to the following formula:

$${\text{y}}_{\text{i}}=\beta_{0}\;+\beta_{1}*age_{i}+\beta_{2}*\;sex_{i}+\beta_{3}*age_{i}*\;sex_{i}\;+b_{1}*ind_{i}\;+{\%}_{\text{i}}.$$

In order to assess possible influences stemming from differences in scanner versions, scanning parameters, including pulse sequence and in-plane resolution, reconstruction methods, and number of previous anesthetic events, additional mixed effect models considering the interaction between these potential confounding variables (pcon ) and age as fixed effects, with fetus as a random effect, were used to analyze differences for volumes and surface metric residuals according to the following formula:

$$\begin{array}{l} {\text{y}}_{\text{i}}=\beta_{0}\;+\beta_{1}*age_{i}\;+\beta_{2}\;*\;pcon_{i}\;+\beta_{3}\;*age_{i}\;*\;pcon_{i}\;+b_{1}\;*ind_{i}\\ \;\;\;\;\;\;\;+{\%}_{\text{i}}. \end{array}$$

Reported p-values for mixed effect models were calculated (Kuznetsova et al., 2017) with the Satterthwaite approximation method and an alpha value of 0.05. All model fits and statistics were carried out using the R software platform (http://www.R-project.org).

## 3 Results

### 3.1 Fetal brain templates

**Fig. 1.**
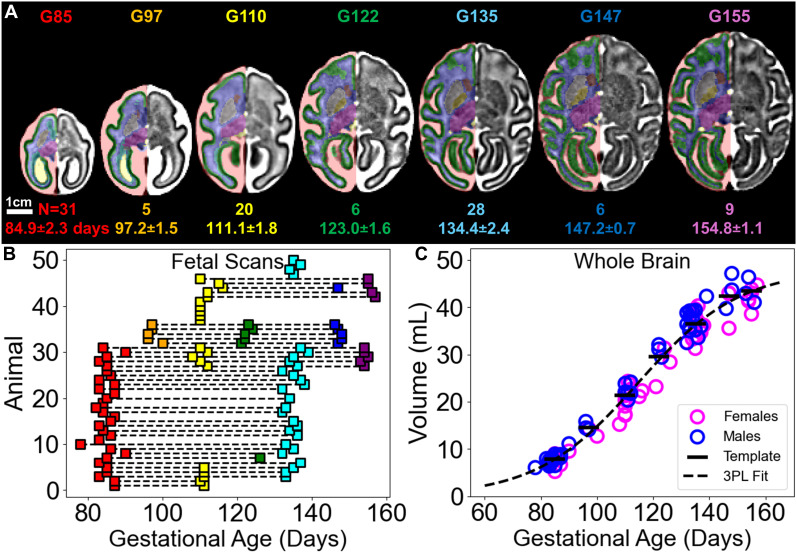
Generation of template images and corresponding segmentations. (A) Mid-axial views of T_2_-weighted template images, with corresponding segmentations overlaid on the right (radiologic view). The number of animals for each gestational age, and the average gestational age for the set of contributing scans, with standard deviations, are included below each image. (B) Each fetus included in this study is represented along the y-axis with scans plotted as squares. Horizontal lines connect repeated scans for each animal. Scan events are color coded by age grouping. (C) Whole brain volumes (parenchyma including ventricles) plotted for females (fuchsia) and males (cyan) as open circles. Template values are included as black horizontal bars. A three-parameter logistic (3PL) expression for the population (ignoring sex) is shown as a dashed curve.

In a sample of 50 (28 females) individual fetuses assessed at 105 imaging time points spanning mid-gestation at G85 to shortly before birth at G155, we characterize fetal brain volumetric development in the rhesus macaque and present 7 fetal age templates with corresponding anatomical parcellations (Fig. 1A).

Templates were constructed from all available nearest-age scans in the sample: G85 from 31 scans (15 females), mean age of 84.9 ± 2.3 days; G97 from 5 scans (2 females), mean age of 97.2 ± 1.5 days; G110 from 20 scans (14 females), mean age of 111.1 ± 1.8 days; G122 from 6 scans (3 females), mean age of 123.0 ± 1.6 days; G135 from 28 scans (14 females), mean age of 134.4 ± 2.4 days; G147 from 6 scans (3 females), mean age of 147.2 ± 0.7 days; and G155 from 9 scans (6 females), mean age of 154.8 ± 1.1 days. Templates were anatomically segmented according to tissue classes described in Table 2. Cross-sectional and longitudinally acquired data were included (Fig. 1B). At G85, near the midpoint of macaque gestation, the average fetal brain volume is 7.8 mL. The brain volume increases to 42.3 mL at the latest assessed time point, G155. This represents an average daily growth rate of 0.49 mL/day over the second half of gestation (Fig. 1C). Considering the 4-year-old adult comparative sample, G155 values corresponds to 60.5% of the adult total brain volume of 69.9 mL. As definitions of “total brain volume” vary due to brain segmentation differences across studies, namely the exclusion or inclusion of extra-axial CSF, the latter more accurately depicting a measure of total intracranial volume (ICV), we additionally report that at G155, fetal macaques reach an ICV of 60.8 mL, corresponding to 65.5% of the adult value of 92.9 mL, in line with similarly parcellated reports of adult rhesus volumes reaching 97.8 mL at 4 years (Malkova et al., 2006) or 95.0 mL at 5 years (Scott et al., 2016).

### 3.2 Gestational volumetric development

**Fig. 2.**
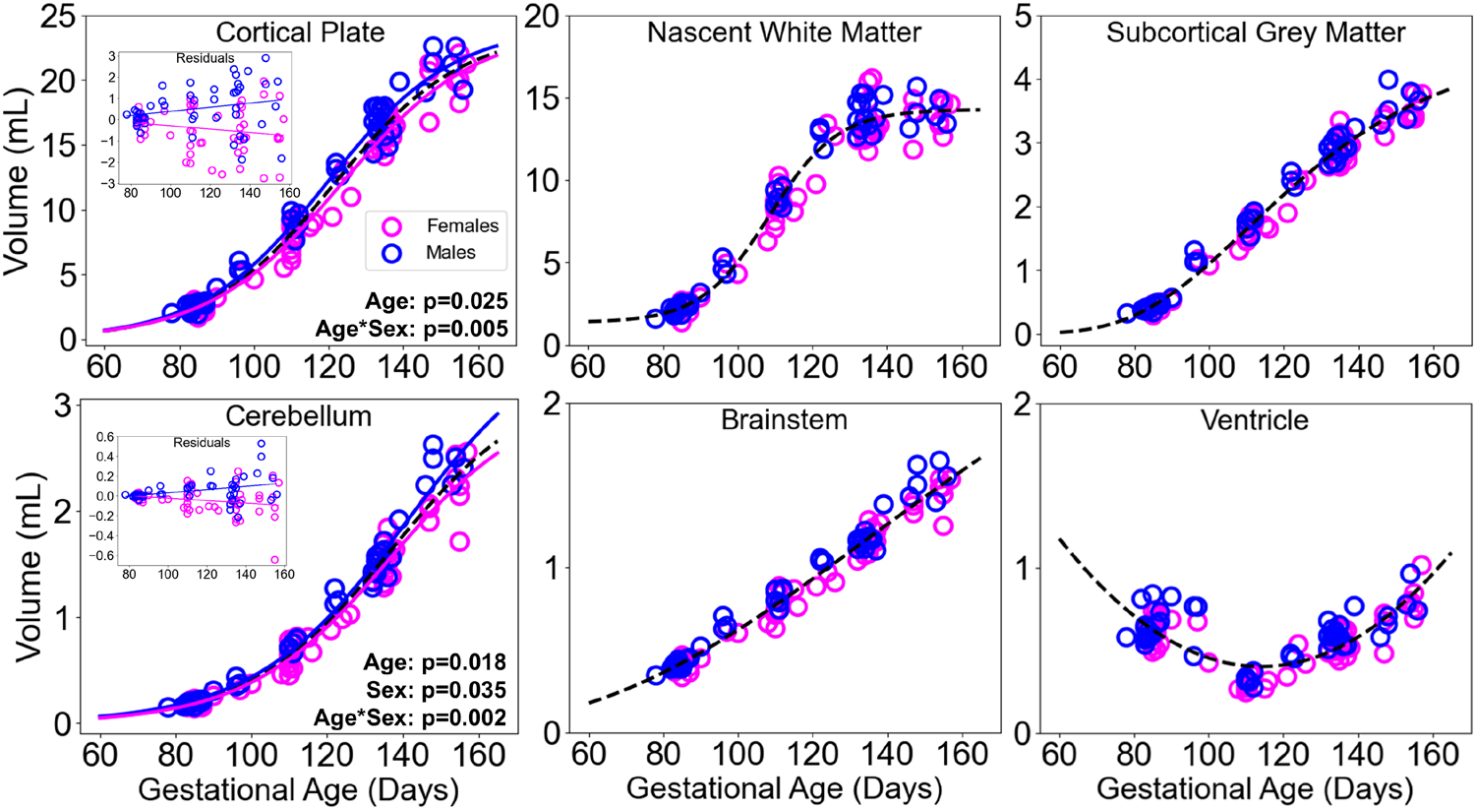
Sexual dimorphism in regional volume development. Cortical plate, nascent white matter (subplate, germinal matrix, and corpus collosum), subcortical gray matter, cerebellum, brainstem, and ventricular volumes plotted for males (blue) and females (fuchsia) with the corresponding selected growth model overlaid for the population (dashed curves). Insets show residuals following nonlinear analysis of age for the cortical plate and cerebellum, which exhibit significant main effects of sex and an age by sex interaction. These regions additionally include separate model fits for males (blue) and females (fuchsia). Linear regressions of the residuals represent the linear mixed effect analyses considering age and sex.

To characterize growth trajectories for different tissue classes, growth was compared between brain regions. The model with maximal likelihood between linear, quadratic, logistic (three and four parameters), and Gompertz models was determined using the BIC (Supplemental Table S2). Whole brain, cortical plate, and cerebellar growth were best approximated by the three-parameter logistic function. Nascent white matter was best approximated with a four-parameter logistic and the Gompertz expression was optimal for representing the brainstem and subcortical gray matter. All regions revealed monotonic increases with age, except for ventricular volume which follows a U-shaped trajectory. Volume decreases from G85 to a minimum at G110, in line with early gestational decreases in ventricular volume observed in humans (Prayer et al., 2006) and other NHPs (Fukunishi et al., 2011), before subsequently increasing and surpassing G85 following G147, and is best fit by a quadratic function (Fig. 2). Early reductions in ventricular volume have been associated with changes in the volume and shape of surrounding parenchymal structures, for example, folding of the calcarine sulcus is coincident with lateral ventricle narrowing and volume reduction (Fukunishi et al., 2011).

For each brain region, potential sex differences were assessed by analyzing linear mixed effect model results applied to the residuals between the selected model and the observed volume for each scan. Analysis of whole brain volume residuals revealed a significant age by sex interaction (β = 0.028, SE = 0.012, p = 0.0199, 95% CI [0.005 0.051]). Comparisons at the regional level revealed a significant main effect of sex for cerebellum (β = -0.21, SE = 0.10, p = 0.035, 95% CI [-0.40 -0.020]). Main effects of age were also observed for the cortical plate (β = -0.011, SE = 0.005, p = 0.025, 95% CI [-0.019 -0.001]) and cerebellum (β = -0.001, SE = 0.0006, p = 0.018, 95% CI [-0.002 -0.0003]), and significant interactions between age and sex for the respective regions (cortical plate: β = 0.02, SE = 0.007, p = 0.0048, 95% CI [0.006 0.032]; cerebellum β = 0.003, SE = 0.0008, p = 0.0020, 95% CI [0.0010 0.0043]) were observed as well. After Bonferroni correction to control for multiple comparisons of regions, only the sex by age interactions for the cortical plate and cerebellum continued to reach significance at an alpha value of 0.05. The interactions illustrate a pattern of males exhibiting increasingly larger brain volumes than females with advancing gestational age, driven primarily by cortical plate volume and to a lesser extent cerebellar volume.

### 3.3 Fetal volumetric neurodevelopmental trajectories

Quantitative indices of fetal growth trajectories are compared between brain regions in Table 3. Separate fits for males and females were used in regions indicating a significant age by sex interaction, and a single population fit was used for regions without an interaction. For the whole brain, cortical plate, and cerebellum, males exhibited higher A values reflecting larger final volumes attained. Maximum growth rates were similar between males and females in the whole brain, with males showing a slightly earlier $t_{mid}$ . In the cerebellum, the larger final volume was achieved through a slower $R_{g}$ occurring at a delayed (later) $t_{mid}\;$ relative to females. Growth in the cortical plate revealed faster maximum growth and earlier $t_{mid}$ , reflecting a more rapid growth than females.

**Table 3.** Fetal growth trajectory parameters.

Region		A	$R_{g}$	$t_{mid}$
Whole brain	M	48.5	0.0566	113
	F	48.0	0.0533	116
Cortical plate	M	24.1	0.0589	120
	F	24.0	0.0561	123
Cerebellum	M	3.78	0.0499	141
	F	3.10	0.0533	137
^a^Nascent white matter		14.3	0.109	108
^b^Subcortical gray matter		4.55	0.0552	111
^b^Brainstem		3.19	0.0166	135

a Growth parameters for nascent white matter are derived from a four-parameter logistic expression.

b Growth parameters for subcortical gray matter and brainstem are a, $R_{g}^{G},$ and $t_{mid}^{G}$, derived from Gompertz expression parameters, as described in the text.

**Fig. 3.**
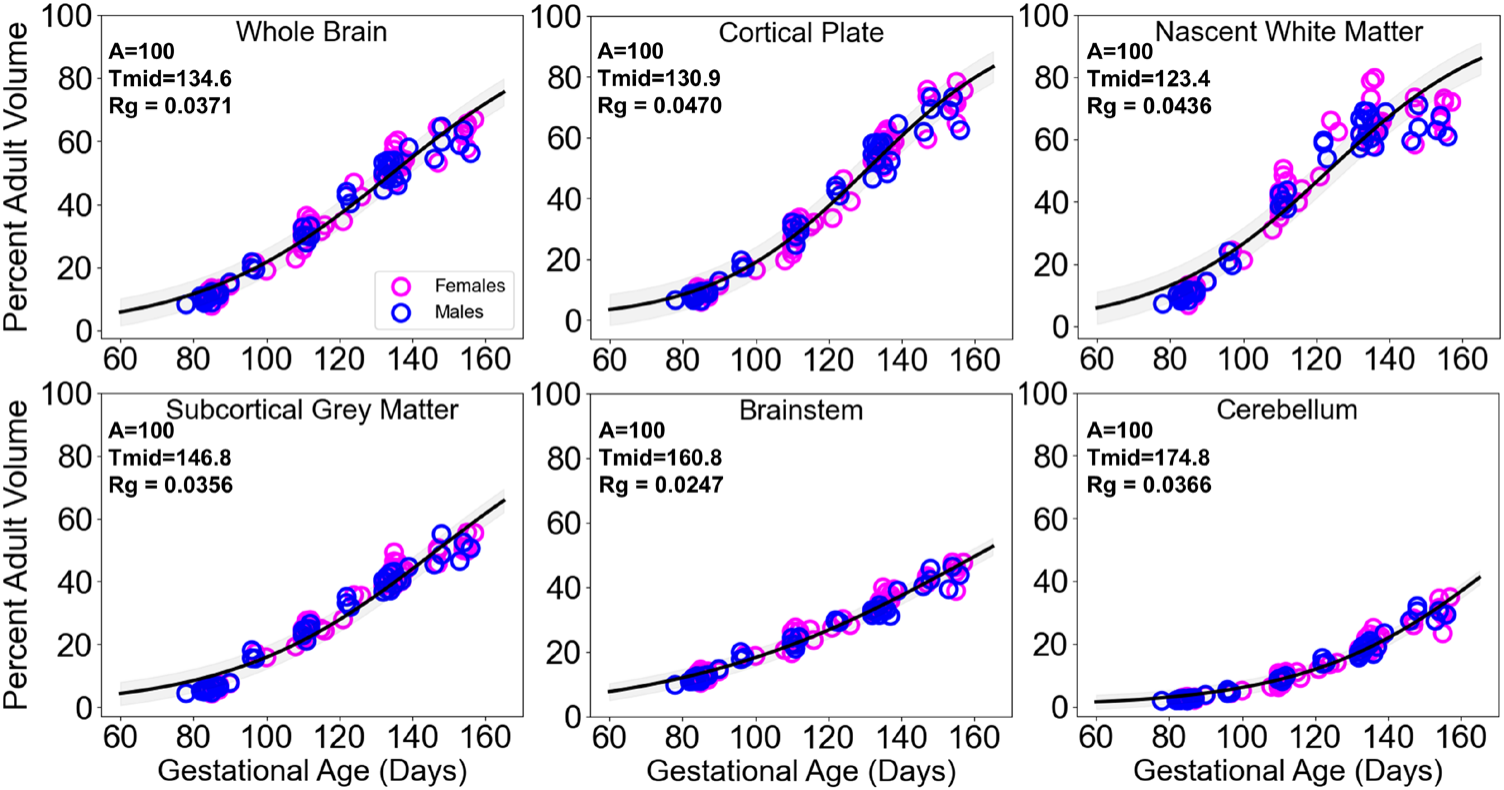
Regional volumes relative to adult sizes. Whole brain, cortical plate, nascent white matter, subcortical gray matter, cerebellum, and brainstem volume plotted for males (blue) and females (fuchsia) as percentages of adult values. Three-parameter logistic fits for all fetuses combined (black) with shading representing 95% confidence intervals. For each region, $t_{mid}$ and $R_{g}$ values are provided for functions constrained at 100% of the adult value (A).

In order to further characterize relative gestational development between brain regions, tissue regional values attained from the adult sample were used to generate fetal volumes as a percentage of the adult values. Fetal-to-adult volume percentages were calculated from post-pubertal male and female regional volume averages and fit with three-parameter logistic expressions constrained to asymptote at 100% of the adult volume for the purpose of comparison. By constraining to the adult volume, fits of fetal volume percentages describe the increases in volume in terms of relative maturity throughout gestation. This procedure reveals a maximum fetal whole brain growth rate of 3.71%/day at G135 (Fig. 3).

Regionally, the maximum growth rate is reached first by the nascent white matter at G123, followed by the cortical plate at G131, the subcortical gray matter at G147, the brainstem at G161, and finally the cerebellum with a theoretical maximum growth rate expected after birth at 175 days post-conception (Fig. 3).

### 3.4 Cortical surface development

**Fig. 4.**
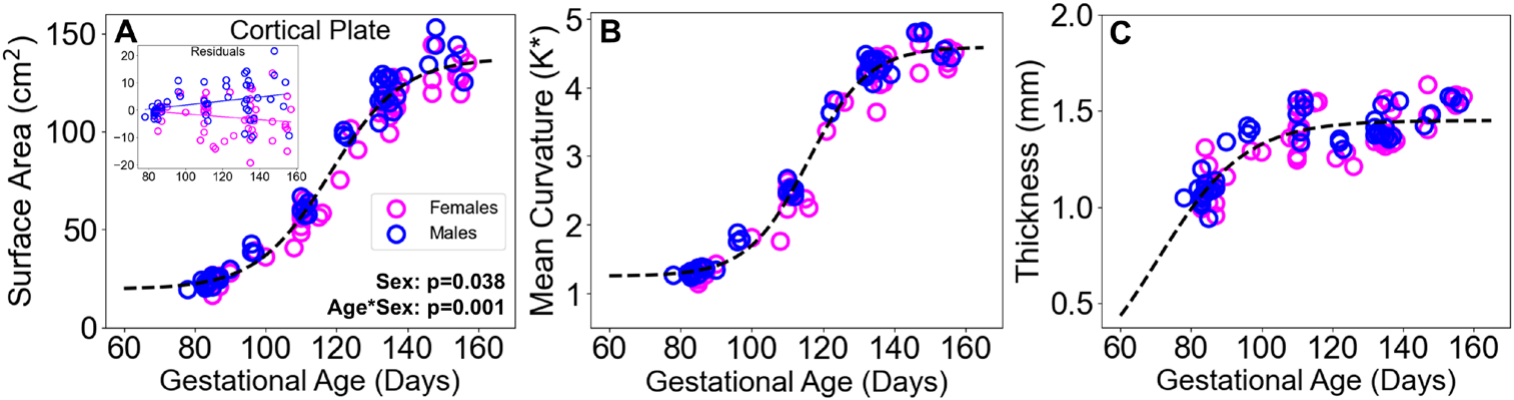
Cortical surface measures. (A) Surface area, (B) nondimensionalized mean curvature, and (C) thickness (cortical volume divided by mid-cortical surface area) are plotted for males (blue) and females (fuchsia) with best-fitting logistic models (black). Linear mixed effects results are shown for surface area in (A), which is the only measure exhibiting a significant interaction.

Growth trajectories of global cortical plate surface area (SA), non-dimensionalized mean curvature ($K^{*}$), and thickness were best fit with a four-parameter logistic function. Linear mixed effect analyses of residuals revealed a significant age by sex interaction (β = 0.12, SE = 0.035, p = 0.0012, 95% CI [0.050 0.19]), as well as a significant main effect of sex for surface area (β = -9.05, SE = 4.30, p = 0.038, 95% CI [-17.41 -0.67]), similar to the pattern of cortical plate volumetric development (Fig. 4). Only the sex by age interaction survived Bonferroni correction for surface measures at an alpha value of 0.05.

Cortical curvature and thickness followed similar trajectories between male and female fetuses. From G85 to G155, average hemispheric surface area increased from 11.9 to 64.8 cm^2^ for females and 11.7 to 67.4 cm^2^ for males. Non-dimensionalized mean curvature increased from 1.31 at G85 to a maximum of 4.67 at G147, nominally higher than the average curvature of 4.45 at G155, representing a late gestational plateau in topological development, while cortical thickness showed more consistent increases across G85 to G155 from 1.10 to 1.56 mm (Fig. 4).

### 3.5 Regional cortical surface expansion

**Fig. 5.**
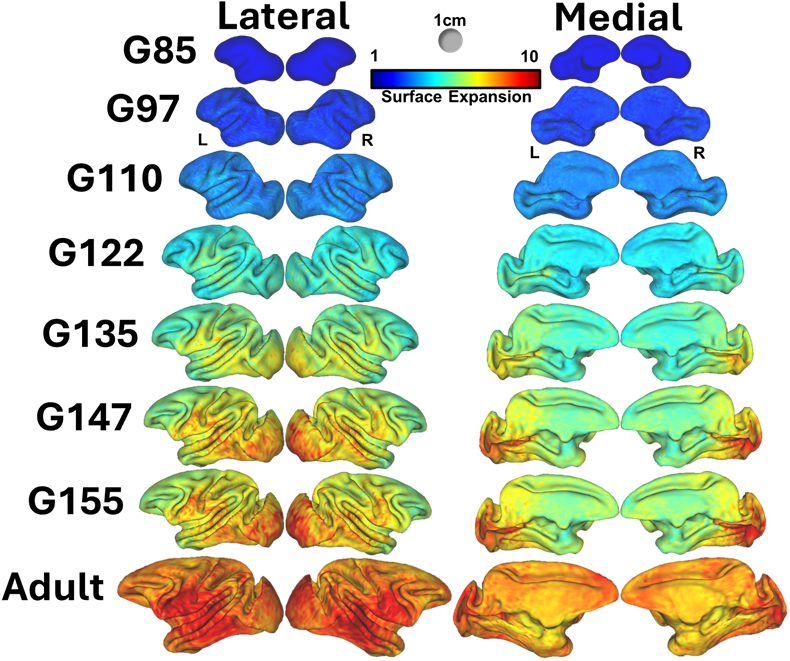
Cortical surface expansion. Node-wise surface expansion maps generated from aMSM analysis on surface atlases from all ages (fetal and adult) to the youngest fetal age (G85). Maps are overlaid on the corresponding age surface. Lateral views of the left and right hemispheres (left) are shown with medial views of the same hemispheres (right). Surface expansion scale ranges from 1 to 10 for all ages, with G85 (no expansion to G85) shown with 0, or no expansion. Scale sphere represents 1 cm^3^.

Following alignment and registration of developmental templates with aMSM, cortical SA expansion from gestational time points to the adult was assessed. Between G85 and adulthood, overall cortical SA exhibited close to 8-fold average increases, with nearly 11-fold expansion evident primarily in the temporal and ventral parietal regions, and to a lesser extent in the occipital lobes (Fig. 5).

Comparing pairwise expansion across all examined ages, G155 to adult exhibited the largest increase of a mean expansion factor difference from G85 of 1.56, with the largest fetal surface expansion occurring between G110 and G122, with a mean expansion difference from G85 of 1.52. Factoring in days between time points, the period between G110 and G122 reveals the greatest and most rapid increase in surface area, aligning with previous examinations of rhesus volumetric expansion (Liu et al., 2020).

**Fig. 6.**
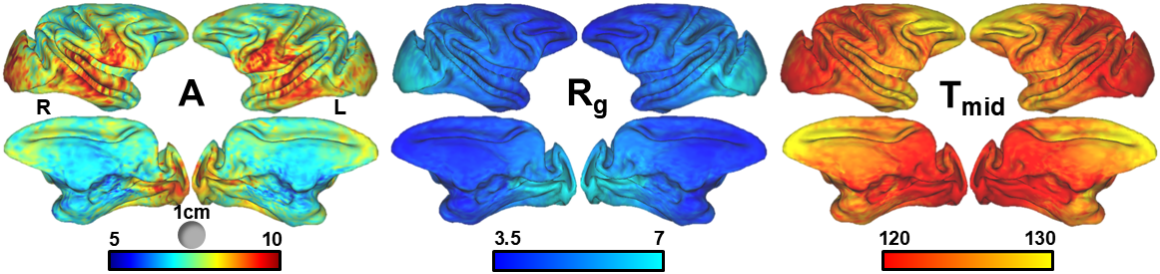
Logistic growth parameters determined at the individual node level. Node-wise surface expansion parameter maps for logistic fits overlaid on adult left and right hemispheres with views of lateral (top) and medial (bottom) surfaces, generated from aMSM registrations between atlas surfaces. Logistic fitting was performed on expansion of nodes A, representing magnitude expansion to the adult volume, $R_{g}$ representing the maximum rate of growth in percent/day, and $t_{mid}$ representing the midpoint in days of maximal growth are shown with corresponding color scales.

Utilizing the node-wise surface expansion coefficients aligned through aMSM, it was possible to fit all nodes representing surface area expansion from G85 to the adult using a three-parameter logistic model to calculate spatially dependent maps of cortical surface area expansion trajectories. Maps of logistic regression parameters are shown in Figure 6.

In agreement with raw surface expansion (Fig. 5), template surface expansion model fits reveal a pattern wherein temporal and ventral parieto-frontal regions exhibit the greatest increases in surface expansion up to a factor of 11, compared with an average of 6–8 for the rest of the cortex. Similarly, large expansions were observed in the medial occipital cortex near the calcarine fissure, with smaller magnitude expansion on the lateral cortex subserving striate cortex. Maximum growth rates reveal a distinct caudal-to-rostral pattern described by rapidly growing (warm) occipital surface, to slowly growing (cool) frontal cortical surface area expansion ranging from about 8 to 2% per day. This caudal-to-rostral pattern of maximal surface area expansion occurring between G120 and G130 generally mirrors the posterior-to-anterior sulcal emergence and development pattern in the macaque (Sawada et al., 2010). However, previous fetal assessments of rhesus macaques did not reveal an association with the development of folding as defined by cortical curvature and surface area expansion (Wang et al., 2017) due to discordance in developmental timing between the two features. The period of maximum growth generally followed the pattern of maximum growth rate, with faster expanding (caudal) regions reaching the $t_{mid}$ earlier in gestation than more slowly expanding (rostral) regions. In terms of overall maturation prior to birth as described by surface area expansion, occipital and medial temporal cortex exhibit the highest relative development with more frontal regions exhibiting the lowest relative maturity with similarly low $R_{g}$ highlighting more protracted development.

### 3.6 Potentially confounding variables

To ensure aspects of data collection do not influence characterization of fetal growth trajectories, we performed a subsequent linear mixed effect analysis on model fit residuals considering the impact of scanner upgrade version and reconstruction technique, scan number (accounting for the influence of multiple anesthetic events for longitudinal measures), acquisition resolution, and pulse sequence. No main effects for these potential confounding influences, nor interactions with age were observed for the whole brain, cortical plate, nascent white matter, subcortical gray matter, brainstem, cerebellum, or brainstem volume (all *p > 0.05*). Similarly, no significant effects were observed for the surface measures SA, K*, or thickness (all *p > 0.05*) (Supplemental Table S3).

## 4 Discussion

Over the second half of the gestational period, the rhesus macaque brain volume expands from 11.2% (7.8 mL) of the brain volume at maturity at G85 to 60.5% (42.3 mL) at G155. Growth of the cerebral cortex is particularly dynamic over this period, as reflected in a 7.9-fold increase in volume of this tissue compartment that arises from an anisotropic 5.6-fold increase in surface area but only 1.4-fold increase in thickness between G85 and G155. Brain shape changes occur to accommodate the mismatch between growth of the cerebral cortex and the rest of the brain. As gyri and sulci emerge, the cortex occupies space that would otherwise be filled by subcortical tissue such as the nascent white matter. Consequently, nascent white matter growth plateaus, while gray matter components of the brain continue to expand.

Previous efforts to quantitatively characterize rhesus brain growth trajectories have faced challenges that stem from the lack of an appropriate analytical model, as well as incomplete sampling of both the accelerating and decelerating phases of growth in gestational development. In prior work, fetal rhesus MRI data were used to approximate growth as different linear rates over defined gestational age ranges (Wang et al., 2017) or between time points in which data were available (Liu et al., 2020). A major limitation of the linear approximation of growth is that the estimated growth rate will strongly depend on the age interval used to measure growth. This limitation is overcome with the sigmoidal modeling approach taken in this study, which enables improved precision for comparisons in growth between brain regions as well as measures in other species. The dataset that was the basis of this study comprehensively assessed the second half of gestation with analysis of 50 fetuses scanned across 105 sessions spanning the G85 to G155 age interval. Measurements from these fetal brain images were referenced to MRI data from a selected set of animals that were an average of 4.5 years old. To model the sigmoidal growth trajectory, time-dependent increases in size were expressed in terms of the timing of transition between accelerating and decelerating growth ($t_{mid}$ ), the rate of maximal expansion ($R_{g}$), and the size of the brain (or brain region) at maturity (A).

### 4.1 Dependence of normative growth on sex and brain region

Sexual differentiation of the macaque fetus begins prior to the first time point measured in this study. The testes of the rhesus macaque begin to produce testosterone by approximately G45 (Resko, 1970), and distinct but varied differences in cord blood testosterone have been observed between male and female macaque fetuses beginning around G60 (Resko et al., 1973). A previous study of fetal macaque brain development did not reveal brain volume differences between male and female fetuses (Liu et al., 2020), despite reports of sexual dimorphism in macaque brain volume early in the postnatal period (Dash et al., 2023; Scott et al., 2016). Similarly, despite extensive evidence of adult brain volume sex differences in humans (Ruigrok et al., 2014), it was not until recently (and with adequate samples) that sexual dimorphism in human fetal brain volume was appreciated with *in utero* MRI (Scott et al., 2011; Studholme et al., 2020). However, head-related measures of fetal sonographic biometry, namely bi-parietal diameter, have long suggested this may be the case (Pedersen, 1980). Thus, the sensitivity of fetal MRI to small effect sizes of sex on fetal brain volume appears to be a limiting factor for early gestation detection of brain volume differences between males and females. The increased sample size and improved analytical modeling of growth in this study provided sufficient sensitivity to observe that the rhesus male brain becomes 0.014 mL larger per day than the female brain over the range of G85 to G155. The presence of this age-dependent difference is similar to findings of MRI investigations of fetal human (Studholme et al., 2020) and ferret (Garcia et al., 2024) brain growth over corresponding periods of development.

Although the overall finding of brain size differences is consistent with observations in other species, further study will be required to understand inconsistencies between species in findings regarding the distribution of specific brain regions that give rise to sex differences, and the manner in which the differences emerge with time. In humans, size differences appear to be driven primarily through differences in nascent white matter volume with only moderate evidence of cortical, and to a lesser extent, cerebellar differences (Studholme et al., 2020). In contrast, for macaques the observed global brain volume differences manifest exclusively in the cortical plate and cerebellar volume. The finding of significant fetal differences in cerebellar volume is somewhat surprising due to the fact that the cerebellum exhibits considerable volumetric development postnatally in both macaques and humans, achieving less than 40% of the total adult value by the latest G155 time point (Fig. 3). However, the gestational period in which cerebellar sexual dimorphism begins to be observed in this study coincides with a period of rapid cerebellar cortical maturity, with greatly increasing molecular layer size and granule cell parallel fiber density in the rhesus macaque (Kornguth et al., 1967). Significant cerebellar volume differences have been observed in human adolescents (Tiemeier et al., 2010) and adults (Raz et al., 2001), but similar comparisons in fetuses (Studholme et al., 2020) and early infants (Gilmore et al., 2007) have yielded only small differences in male and female cerebellar volumes. A highly similar MRI analysis of brain growth performed on ferrets also revealed sex-dependent development (Garcia et al., 2024). In developing ferrets, males have larger brains, but this is achieved through more protracted growth, which occurs at a similar rate, resulting in a similar $R_{g}$, but larger $t_{mid}$ and A values. In this study, differences in rhesus macaque brains appear to arise from more rapid growth of the brain, with similar $t_{mid}$ values in both sexes. It is difficult to conclude whether the observed differences in regional distribution and timing truly reflect species differences, or whether they arise from differences in experimental approaches, such as image segmentation procedures applied to human fetal data, or experimental design and time periods sampled in the ferret data. The common primary finding, that brain size differences between males and females emerge over the developmental period, is consistent between human, rhesus macaques, and ferrets.

### 4.2 Regional patterns of cerebral cortical surface area expansion

Surface-based registration techniques have been used to assess whether neocortical surface area expansion is uniform or whether it possesses structured regional variation in ferrets (Garcia et al., 2024, 2025), rhesus monkeys (Wang et al., 2017), baboons (Kroenke et al., 2007), and humans (Garcia, Robinson*,* et al., 2018). The combination of the automated surface registration algorithm aMSM (Garcia, Robinson*,* et al., 2018; Robinson et al., 2018) with dense, systematic sampling of the growth phase of cortical development has recently enabled a highly detailed analysis of regional patterns of ferret cortex surface area expansion to be performed (Garcia et al., 2024). As was done in this study (Fig. 6), the surface area associated with each surface vertex was fitted to a logistic equation, resulting in the generation of cortical maps of A, $R_{g}$, and $t_{mid}$ .

In the ferret, the $t_{mid\;}$ map was observed to be highly similar to the pattern of water diffusion anisotropy within cerebral cortical gray matter (Garcia et al., 2024; Kroenke et al., 2009). Gray matter diffusion anisotropy in the developing cortex has been proposed to relate to development of the neuropil. In the undifferentiated cortex, diffusion anisotropy is high due to the presence of dense neural and glial processes that are uniformly oriented in radial directions. Obliquely oriented axonal and dendritic collaterals are generated during maturation, and this structural heterogeneity is associated with developmental reductions in water diffusion anisotropy (Kroenke, 2018). In several mammalian species including ferrets (Garcia et al., 2024; Kroenke et al., 2009) and nonhuman primates (Kroenke et al., 2007; Wang et al., 2017), a regional pattern of cortical diffusion anisotropy parallels regional patterns in the age of neurons due to a transverse neurogenic gradient (TNG) (McSherry, 1984; Sidman & Rakic, 1973; Takahashi et al., 1999). Thus, the observed close association in ferrets between the $t_{mid}$ pattern for surface area expansion and cortical diffusion anisotropy, with regions characterized by early $t_{mid}\;$ also being regions with relatively early reductions in diffusion anisotropy, supported the interpretation that surface area expansion follows the developmental gradient established by the TNG (Garcia et al., 2024; Knutsen et al., 2013).

Comparisons between cortical diffusion anisotropy and surface area expansion patterns in NHPs prior to this study have been equivocal, but results shown in Figures 5 and 6 reveal conclusive differences between regional patterns of SA expansion and previous analyses of diffusion anisotropy. Surface-based registrations that utilized manually drawn landmarks were used to identify differences between rostral and caudal halves of the brain in the baboon (Kroenke et al., 2007), and have demonstrated variation throughout the rhesus macaque cortex (Wang et al., 2017). However, in addition to the reliance on manually placed constraints in the registration procedures, both of these studies examined limited developmental time points, which hindered the ability to assess whether the extent of surface area expansion is due to the nonlinear growth trajectory described above. In this study, the robust aMSM registration method combined with dense sampling of growth clearly demonstrates that the $t_{mid}$ pattern is dissimilar to regional diffusion anisotropy in the rhesus macaque. The earliest $t_{mid}$ values in Figure 6 of approximately 115 days gestation reside on the occipital lobe. This region is among the latest to experience reductions in water diffusion anisotropy baboons (Kroenke et al., 2007) and rhesus macaques (Wang et al., 2017) due to its distance from the source of the TNG, which is near the insula. The precentral gyrus is the region that most rapidly loses cortical diffusion anisotropy in nonhuman primates, but the $t_{mid}$ value for surface area expansion is in the middle of the range shown in Figure 6 of approximately 125 days gestation. Interestingly, a commonality between the $t_{mid}$ pattern of Figure 6 is observed with extent of synaptic development found by electron microscopy (Granger et al., 1995). Primary sensory and motor cortical regions and primary visual cortex were found to develop earlier than cortical regions on the lateral and medial prefrontal lobe. The rank order of $t_{mid}$ values follows the reported extent of synaptogenesis (Granger et al., 1995). Taken together, the regional pattern of surface area expansion is distinct from the TNG and pattern of cortical diffusion anisotropy in rhesus macaques, but surface area expansion may still be related to regional patterns in synaptogenesis.

The remaining two logistic equation parameters, A and $R_{g}$, showed several similarities to the parameters reported for the ferret, which is the only other animal species to have undergone similar analysis of regional SA expansion. Substantial variation in the extent of surface area expansion is apparent in the map of A (Fig. 6), ranging from a factor of 6 in the frontal lateral and medial cortex to approximately 10 in the ventral lateral frontal lobe and temporal cortex. In ferrets, the region with the highest A parameter was found in the occipital cortex, and in rhesus, relatively high values of 9 to 10 were observed in the occipital lobe. Regional patterns of maximal growth rate were also found to be similar between rhesus macaques and ferrets. Specifically, prefrontal cortex exhibits the lowest $R_{g}$, and occipital cortex is the highest in both species. Work in additional species such as humans will be valuable for establishing the extent of conservation of these patterns, and lead to insights into potential underlying developmental processes that give rise to variation in A and $R_{g}$.

## 5 Conclusion

Anatomically segmented T_2_-weighted templates were generated from 105 *in utero* brain scans of 50 (28 female) fetal rhesus macaques at G85, G97, G110, G122, G135, G147, and G155, as well as a corresponding 4.4-year-old adult template generated from 20 (10 female) macaques. These templates along with mid-cortical template surfaces at the adult and fetal ages are provided as a resource for the primate neuroimaging community for the study of rhesus macaque volumetric and cortical morphological neurodevelopment. Using this unique resource of *in utero* scans, we characterize fetal volumetric and surface growth trajectories, with fetal whole brain volumes achieving 60.5% of the young adult value at G155, shortly before birth. Surface area expansion patterns in the fetal macaque brain were found to be more consistent with patterns of synaptogenesis than structural neuropil maturation patterns as described by the TNG. We also report evidence of sexual dimorphism in macaque brain volume and surface area, driven primarily by increasing large values for males than for females in the cortical plate and cerebellum.

## Supplementary Material

Supplementary Table 1

Supplementary Table 2

Supplementary Table 3

## Data Availability

Data are available as the ONPRC Fetal Macaque Brain Atlas 2.0 at https://www.nitrc.org.
